# Role of Substance P in the Pathophysiology of Inflammatory Bowel Disease and Its Correlation With the Degree of Inflammation

**DOI:** 10.7759/cureus.11027

**Published:** 2020-10-18

**Authors:** Mauli Patel, Sharathshiva Valaiyaduppu Subas, Mohammad R Ghani, Vishal Busa, Ahmed Dardeir, Suganya Marudhai, Ivan Cancarevic

**Affiliations:** 1 Internal Medicine, California Institute of Behavioral Neurosciences & Psychology, Fairfield, USA; 2 Neurology, California Institute of Behavioral Neurosciences & Psychology, Fairfield, USA; 3 Internal Medicine/Family Medicine, California Institute of Behavioral Neurosciences & Psychology, Fairfield, USA

**Keywords:** inflammatory bowel disease, substance p, neuropeptides, ulcerative colitis, crohn’s disease

## Abstract

Inflammatory bowel disease (IBD) is a multi-factorial, chronic inflammation of the gastrointestinal tract, containing ulcerative colitis (UC) and Crohn's disease (CD). In UC, inflammation and sores are confined morphologically and microscopically to the mucosa, the innermost surface of the colon and the rectum. Although, in CD, the infection is granulomatous and transmural, affecting the entire gastrointestinal tract from the mouth to the anus, with the skip area in-between. A Neuropeptide, substance P (SP), which acts as a neurotransmitter and as a neuromodulator, plays a vital role in the brain-gut axis under stress. Owing to the pro-inflammatory effects of SP, neuropeptide dysregulation induces inflammation in the intestine. There are variations in the distribution of substance P immunoreactive fibres in the various intestinal layers. The highest concentration of SP is in the mucosa and the lowest concentration in the lamina propria of the intestinal muscular membrane. Reduced vasoactive intestinal peptide (VIP) levels and elevated SP levels observed in the colonic mucosa of IBD by using immunohistochemistry and immunoassay. This literature review aims to find out the correlations between the level of substance P (SP) and disease activity. We conducted a literature review on IBD, SP, and we searched PubMed and Google Scholar for relevant articles in English. The result of the study supports a positive relationship between the level of substance P (SP) and disease activity, with increased concentration of substance p in the colon and rectum of CD and UC patients. It is concluded that patients with active CD, along with inflammatory changes, had elevated plasma SP levels and immunoreactivity of SP in the colon than those seen in control and inactive cases. These alterations are more prevalent in ulcerative colitis than Crohn's disease and are more prevalent in the moderately infected area than the least affected area of the intestine.

## Introduction and background

Inflammatory bowel disease (IBD) is a chronic gastrointestinal tract inflammation with the relapsing and remitting course. IBD consists of Ulcerative colitis (UC), Crohn's disease (CD), and Microscopic Colitis (MC). Patients with IBD begin to show symptoms in their 20s that persist throughout their lifetime, increasing the risk of colon cancer in UC and CD. However, MC is an older age disease [1]. Family history, environment, dietary changes, excessive use of antibiotics are some of the factors that lead to IBD [2]. Dysregulation of the intestinal immune system in response to changes in the intestinal flora contributes to important pathological factors [3]. Ulcerative colitis and Crohn's disease have many distinguishing clinical presentations and pathological characteristics [3,4]. Bloody diarrhoea, colicky abdominal pain, and tenesmus are more common in ulcerative colitis (UC) [4].

On the other hand, mucoidal diarrhoea and systemic features such as anorexia, malaise, and fever are far more common in Crohn's disease (CD) [4]. In Ulcerative colitis (UC), inflammation and sores are confined only to superficial intestinal layers. In most cases, it involves the large intestine and the rectum, and the inflammation is constant. However, in Crohn's disease (CD), the inflammation extends from the mouth to the anus with skip areas in-between. In CD, inflammation is granulomatous and associated with more complications, such as enterovesical fistula, strictures, abscess, perianal disease as compared to UC [5]. IBD can present with extraintestinal symptoms that involve skin, eye, joints, kidney, liver, biliary, and vascular tracts [4]. Clinical presentation and combination of radiological, endoscopic, and histological reports required for diagnosis. Since inflammation is the core pathophysiological process behind IBD, 5- Aminosalicylic acid (5-ASA) is the main treatment modality [3]. Another treatment modality is surgery, which is not curative but minimizes the impact of the disease. Studies suggest that 50% of patients require surgery within ten years of the diagnosis, and 70-80% of patients require surgery in their lifetime [6].

IBD is considered a developed-country illness affecting over three million people in the USA and Europe. The studies indicate that the incidence in North America, Oceana, and several European countries is estimated to surpass 0.3. Overall, the age-standardized prevalence rate had grown from 79.5 per 100,000 population in 1990 to 84.3 in 2017. The USA had the highest age-standardized prevalence rate at the national level, 464·5 per 100 000 population, followed by the UK at 449·6 per 100 000 population. The Caribbean has reported the least age-related prevalence rate at 6.7 per 100,000 [7]. The age-standardized mortality rate decreased from 0·61 per 100,000 in 1990 to 0·51 per 100,000 in 2017 [7,8]. Consequently, IBD has a significant influence on the quality of life and economy of the community.

The intestine is the critical regulating organ of the immune system in the body since it has a rich peptidergic innervation. Neuropeptide substance P (SP) acts as a neurotransmitter and a neuromodulator. It extends across all intestinal layers, including submucosa and myenteric plexus [9]. SP modulates immunological, vascular, and motor phenomena in the intestines [10-12]. Substance P also functions as a pro-inflammatory mediator released from sensory nerves, myenteric neurons, and inflammatory cells such as lamina propria eosinophils. As a result, intestinal inflammation stimulates the brain-gut axis and releases substance P, which binds with high affinity to neurokinin 1 receptor (NK1R) expressed on nerves, epithelial cells, and immune cells such as mast cells, macrophages, and T cells [10]. Activation of these cells releases cytokines and chemokines that modulate diarrhoea, inflammation, and intestinal motility [10,12]. IL-12 stimulates the release of substance P. However, IL-10 and TGFβ inhibit substance P production from T cell and macrophage, respectively [13].

IBD has a greater impact on the social economy. Therefore, in this review article, our primary focus will be on what is the function and role of neuropeptide substance P (SP) in IBD and what is the relationship and association between the level of substance P (SP) and the extent of inflammation in IBD.

## Review

Role of the enteric nervous system and immune system in the gastrointestinal tract

The gastrointestinal tract neuroendocrine regulatory system (NES) is split into two main parts, the gastrointestinal (GI) tract endocrine cell and Enteric Nervous System (ENS). Endocrine cells are present in all parts of the gastrointestinal tract from mouth to anus except for the esophagus [14-16]. These cells are located between the mucosal epithelial cells that contain hormonal peptides and amines in an enormous amount [17,18]. The endocrine GI tract cells have all the essential components for both afferent and efferent synaptic transmission [14]. GI tract has a distinct, separate nervous system: one in the submucosa (submucosal plexus), and another between muscles (myenteric plexus). Afferent and Efferent nerve fibres of the central nervous system (CNS) and autonomic nervous system (ANS) modulate neurons of the ENS [14,19]. Studies reveal that there is an increase in density and proportion of GI tract endocrine cells in CD and UC samples of patients and animal models of human IBD [14]. The extent of the alteration depends on the type of disease (CD v/s UC), the affected area of the intestinal wall, and mucosal inflammatory behaviour [20]. Based on this information, we can conclude that changes in the enteric nervous system are responsible for the pathophysiological changes of IBD and their associated symptoms.

Substance P, a neuropeptide, is a member of the tachykinin family. Substance P-containing neurons distribute throughout the intestinal wall. It is located in enteric efferent neurons and expressed by several immune cells (IC), including T cells, macrophages, dendritic cells, and eosinophil cells [21-23]. Substance P is responsible for the migration of innate IC, such as neutrophils and macrophages, and adaptive IC such as T lymphocytes [24]. Moreover, substance P stimulates the proliferation of lymphocytes and modulates the behaviour of innate and adaptive IC [25]. Substance P is, therefore, known to be one of the vital pro-inflammatory mediators in the GI tract. Mathison et al. showed that SP regulates neutrophil aggregation by enhancing the activation of other inflammatory factors, such as leukotriene B4, neutrophil adhesion activating platelet factors, and migration and biochemical reactivity [26].

The intestinal motility is controlled by non-adrenergic non-cholinergic neurons (NANC), along with adrenergic and cholinergic neurons [27,28]. NANC neurons have excitatory and inhibitory components. The studies specified that NANC inhibitory nerves were more prevalent than NANC excitatory nerves in the control of enteric nerve function in both normal colon and colon of patients with chronic ulcerative colitis [28]. The research by Snape et al. confirmed that inflammatory changes in the intestines are associated with changes in the structure of the ENS, the neurotransmitter, and its receptors [29]. Tomita et al. (1998, 2000) also agree with this fact [30]. Goldin et al. (1989) reported that substance P (SP) is a part of NANC excitatory neurons and its level significantly increases in chronic ulcerative colitis (CUC) [31]. Stoyanova et al. also mentioned that gastrointestinal motility dysfunction, including UC, is associated with dysfunction of the enteric nervous system in the colon [25]. Based on all the evidence, we can believe that the GI tract's immune dysfunction is the primary pathology behind IBD.

Three receptors have governed the action of SP: NK-1, NK-2, and NK-3, all of which belong to the family of G-protein receptors [32]. SP has the highest affinity for NK-1 receptors and low affinity for NK-2 and NK-3 receptors [32]. In certain studies, results of biopsy samples from UC and CD demonstrate an elevated level of receptor binding site for SP in arterioles, venules, and lymph nodules as well as enteric neurons, submucosal blood vessels [33]. Hence this suggests that the SP-NK-1 receptor system might represent a significant immunoregulatory system involved in IBD.

Renzi D. et al. provided evidence in his article that the NK-1 receptor and its natural ligand SP affect the immune response in the human intestine and liver. A seven-fold increase in NK-1 receptor transcripts in the CD colon and a modest increase in UC compared to healthy colon was observed in one research [33]. Kimura M et al. added that, in IBD patients, decreased levels of VIP in the mucosa and high density of SP-receptors in the germinal centre of the lymph nodes were found respectively [34]. Therefore, it is speculated that VIP and SP have immunoregulatory functions in the colonic mucosa. The distribution abnormality of VIP and SP neurons results in the immunological dysfunction of the mucosa of patients with IBD and leads to chronic IBD.

Distribution of substance P in IBD and normal colonic mucosa

Numerous studies have established that immunological imbalances in the colon are the primary pathological cause behind IBD. Few types of research, therefore, concentrate on the distribution of nerve fibres of distinct neuropeptides in the healthy colon and colonic IBD samples. The main component of the intestinal neurons, neuron-containing SP, is distributed to the mucosal epithelium, the mucosal-submucosal blood vessels, and the muscular layer. Pälvi Vento et al. recorded differences in the distribution of immunoreactive fibres of substance P across different intestinal layers. In the case of a healthy colon, submucosa and mucosa have thick substance P immunoreactive fibre bundles as well as thin substance P around the blood vessels. On the other hand, Lamina propria has the least amount of substance P immunoreactive fibres and, the muscular layer has thin SP immunoreactive fibres run parallel to muscle fibres. The density of immunoreactive fibres remains constant for mucosa, submucosa, and muscular layer. Samples from the moderately affected UC colon, indicating an increase in the intensity and density of the immunoreactive nerve fibres of substance P in lamina propria. Yet, there is no increase in the density of immunoreactive fibres due to intense inflammation and the lack of villi/no villi in the extremely affected area. As a result, the analysis concluded that the sensitivity of immunoreactive fibres improved in the mild to moderate UC range [35].

Kimura et al. mentioned that both VIP and SP-nerves increased in hypervascular areas and decreased depending on the extent of inflammation. Additionally, the concentration of substance P in infected colonic mucosa reduced when expressed per gram of wet weight, although no substantial difference observed when expressed per gram of extracted protein. The study showed histopathological characteristics of IBD mucosa that rely on the degree and pattern of distribution abnormalities of the neuropeptide nerve fibre [34]. The outcome of the Korman et al. research differs from other studies because they find SP binding sites only in the muscular layer rather than in the mucosal layer of the human intestine [36]. The density of SP immunoreactive fibres was localized five out of eighteen in control, four out of nine in CD, and five out of nine in the UC specimen. The result of the study showed that there was no significant difference in the area density of nerve-containing neuropeptide in the colonic mucosa of UC and CD patients [37]. We must say, the distribution abnormalities of neuropeptides present in different layers of the intestine of IBD, and the severity of the distribution abnormality depends on the level of inflammation. In the mildly affected area, the less distribution abnormality is found relative to the moderately affected area.

Correlation between the level of substance P and degree of inflammation in IBD patients

This study indicates that the degree of inflammation is proportional to the change in SP immunoreactivity when the control specimens and the least to moderately affected UC specimens are related. It proposed that the effect of UC on the substance P immunoreactivity could be much greater than that demonstrated by the present information due to the absence of anti-inflammatory drugs in the control system. The overall density of substance P-immunoreactive nerve fibres in lamina propria was 0.55 ±0.15, 1.30 ±0.35, 2.22 ±0.28 in the normal colon, the least affected UC colon (p=0.087), and the moderately affected UC colon (p<0.001), respectively. The density of immunoreactive fibres in moderately and least affected colon in UC was greater than the control value by 136% and 304% respectively. The difference between the value of the least affected and moderately affected UC colon was significant (p<0.05). The value in moderately affected UC colon was 71% greater than that for the least affected UC colon. The total area of substance P-immunoreactive nerve fibres were 8±2 in the normal colon, 17±5 in the least affected UC colon (p<0.12), and 30±4 in moderately affected UC colon (p<0.001). The values of the least affected UC colon and moderately affected UC colon were 114% and 280% higher than the control value. Besides, the value of moderately affected UC colon was 76% greater than that for the least affected UC colon [35]. These findings demonstrated that the density of SP fibres increased significantly in active cases of UC compared to inactive cases (P < 0.01) [12]. Although, these researchers noted that patients receiving high doses (> 10,000 mg) of steroid (prednisone) demonstrated a significant high linear density of immunoreactive fibres compared to those receiving low doses (< 10,000 mg) of steroid in active cases of UC [12]. Sjölund et al. analyzed the concentration of neuropeptides in the intestine by using an immunocytochemical study, which observed only a few changes in the concentration compared to the control group. Hence, the result of this study differs from previous studies [38]. Conversely, the study of Mazumdar et al. supports the correlation between the level of substance P (SP) and disease activity by noticing increased concentration of SP in tissue samples from the colon and rectum of CD and UC patients [9] [11,39,40]. Francesca et al. in 2011 established that patients with active CD along with inflammatory changes demonstrated a high serum SP level than those found in control (P<0.001) and inactive cases. Interestingly, they did not find any discrepancy in the level of serum neuropeptide in complicated CD samples such as strictures, fistula, and abscess as compared to noncomplicated active CD cases [20]. However, they found high serum SP levels in an active case of IBD compared to control (P=0.001). On the other hand, Renzi et al. mentioned that the concentration of SP in both active and inactive UC patients were not different from those reported in healthy controls through a radioimmunoassay [33]. After all the studies have been analyzed, we may assume that the degree of inflammation changes the concentration of neuropeptides in various intestinal regions. The area with maximum changes in IBD was the mucosa layer of the moderately affected colon. The least improvements in SP immunoreactivity are seen in extreme inflammatory regions due to damage to all inflammatory cells and the structure of the intestine. Table 1 shows the conclusion of some of the studies indicate that substance P plays a significant role in IBD.

**Table 1:** This table focuses on the results/conclusion of a variety of significant research on similar topics. SP- Substance P, UC- Ulcerative Colitis, IBD- Inflammatory Bowel Disease, VIP -Vasoactive Intestinal Peptide, RIA-Radioimmunoassay

Name of authors	Study method	Result/conclusion
Mitsuo Kimura [34]	immunohistochemistry	In control: SP-nerves were fine and distributed in the lamina propria. In UC: Although the changes of SP-nerve distribution in UC were less obvious than those of VIP-containing-nerves decreased in severe inflammatory lesions of active UC.
Pälvi Vento, Tuula Kiviluoto [35]	Immunohistochemistry	The increased density of substance P fibres in UC is due to increased peptide synthesis, and as a result, increased visibility of the fibers by immunohistochemistry.
Toshiaki Watanabe [12]	Immunohistochemistry	The study found substance P fibres in lamina propria of IBD patients. The linear density of substance P fibres substantially increased in active cases compared to inactive.
Ahmed Metwali [41]	RIA, Insitu hybridization, Immunohistochemistry	The results of this study show that lamina propria inflammatory cells from most patients with IBD and some normal controls store authentic SP and VIP.
Hua XY [42]	Immunohistochemistry	Mast cells are regulated by the nervous system and the SP is responsible for the degranulation of mast cells.

Limitation

In this traditional review article, we were not able to identify any studies written after 2015, and we did not perform any quality assessment of individual studies. There is insufficient information about how the degree of SP affects the course of the disease and its role in the prognosis.

## Conclusions

In this traditional review article, our main focus was to examine the role of the neuropeptide substance P (SP) in the pathogenesis of inflammatory bowel disease (IBD) and to analyze whether or not there are a correlation and causation between the level of the substance P (SP) and the degree of inflammation in IBD. In our research, we find that dysregulation and imbalance of neuropeptides, primarily substance P, is responsible for inflammatory changes in IBD. Increased serum substance P level and the density of substance P immunoreactive fibres were observed in the lamina propria of IBD patients. Besides, we notice higher concentrations of SP immunoreactive fibres in patients with Ulcerative colitis as compared to Crohn's disease due to a higher degree of inflammation. So, we can say that there is a strong correlation between SP and the degree of inflammation. Also, variations in the strength and density of SP containing neurons in colonic mucosa are prominent in moderate inflammation compared to mild inflammation. Hence, we conclude that there is a significant positive linear correlation between the level of substance P (SP) and the extent of inflammation in IBD. Further research on this subject is required due to uncertainty as to how corticosteroids influence the level of substance P (SP) and how the SP level fluctuates in the IBD subgroup.
